# Comparative Analysis of the Composition of Exosome-like Nanoparticles from Dried and Fresh *Portulaca oleracea* L.

**DOI:** 10.3390/molecules30244715

**Published:** 2025-12-09

**Authors:** Yinjie Ma, Kaige Yang, Hai Hu, Wenchang Fu, Ce Li, Yutong Zeng, Xinyan Li, Yan Wang

**Affiliations:** School of Pharmacy, Shanghai Jiao Tong University, Shanghai 200240, China; bubble1205@sjtu.edu.cn (Y.M.); ykg19951015@sjtu.edu.cn (K.Y.); huhaihh@sjtu.edu.cn (H.H.); wenchangfu333@163.com (W.F.); sjdxlice@sjtu.edu.cn (C.L.); zeng_yt@sjtu.edu.cn (Y.Z.)

**Keywords:** exosome-like nanoparticles, *Portulaca oleracea* L., extraction and separation, multi-omics analysis, dried and fresh states

## Abstract

Plant-derived extracellular vesicles (PEVs) have emerged as a promising area of research in biotechnology with enormous potential in drug delivery, skincare, and functional foods. Currently, PEVs are obtained primarily from fresh and dried materials through soaking and extraction; however, little is known about the differences in their contents. Using *Portulaca oleracea* L. as the research object, this study firstly employed a method that combined differential and ultracentrifugation with membrane filtration to separate and purify exosome-like nanoparticles from dried material (D-PELNs) and fresh material (F-PELNs). Then, multi-omics analysis compared the small-molecule metabolites, lipid profiles, and protein expression patterns. Both D-PELNs and F-PELNs showed typical cup-shaped morphology, with mean particle sizes of 139 nm and 186 nm, and mean zeta potentials of −16.015 ± 0.335 mV and −6.29 ± 0.19 mV, respectively. Both types contained diverse small-molecule metabolites. Among them, terpenoids (e.g., caesaldekarin e) were more abundant in F-PELNs, whereas carboxylic acids and their derivatives (e.g., citric acid) were predominantly found in D-PELNs. Both types had abundant lipids. D-PELNs exhibited greater lipid diversity than F-PELNs, with notable enrichment in phosphatidylcholine (18.48%) and ceramide (17.02%). F-PELNs mainly consisted of functional neutral lipids, such as monoglycerides and triglycerides. Proteins involved in plant morphogenesis and secondary-metabolite biosynthesis were also identified. Proteins from both *Portulaca oleracea* L.-derived exosome-like nanoparticles (PELNs) were localized to intracellular structures, including the cytoplasm and mitochondria of the cells. D-PELNs had a higher protein content related to carbon metabolism, whereas F-PELNs were more enriched in proteins related to secondary metabolite synthesis. In summary, D-PELNs and F-PELNs were successfully isolated and characterized, and their compositions were analyzed and compared using multi-omics approaches. These findings identify the specific chemical components of PELNs and offer new insights for comparing the compositional differences between exosome-like nanoparticles derived from dried and fresh plant states.

## 1. Introduction

Plant-derived extracellular vesicles (PEVs) show great promise in biomedicine and functional foods as carriers of natural bioactive substances. Among these, exosome-like nanoparticles (ELNs) have emerged as a frontier hotspot in nanobiomedicine due to their stable lipid bilayer, good biocompatibility, low immunogenicity, and ability to cross species barriers. ELNs are lipid bilayer vesicles with diameters ranging from 30 to 150 nm that contain proteins, lipids, nucleic acids, and small-molecule bioactive compounds [1]. ELNs, which were first isolated from sunflower seedlings in 2009, have been shown to play crucial roles in multiple physiological processes. These include immune regulation (e.g., *Portulaca oleracea* L.-derived ELNs (PELNs) alleviate colitis by promoting CD4+CD8+ T cell expansion), redox balance (e.g., grapefruit ELNs eliminate reactive oxygen species), and tissue regeneration (e.g., strawberry ELNs increase fibroblast migration speed by 2.3-fold), among others [2,3].

The comprehensive characterization of the complex cargo within plant-derived ELNs is important to understanding their biological functions. To this end, high-throughput omics technologies, including metabolomics, lipidomics, and proteomics, have become indispensable tools. These approaches enable the simultaneous identification and quantification of numerous biomolecules, thereby providing a holistic view of the plant-derived ELNs’ composition. For instance, multi-omics analyses have been successfully applied to plant-derived ELNs from saffron tepals and turmeric, revealing species-specific bioactive components and offering insights into their biological functions and potential roles in cross-kingdom communication [4,5].

*Portulaca oleracea* L. is a traditional medicinal and edible plant rich in bioactive compounds, such as flavonoids, organic acids, polysaccharides, and unsaturated fatty acids. It has been found to have a variety of physiological functions, including antioxidant, anti-inflammatory, and immunomodulatory effects [6,7]. According to a 2023 study, oral administration of PELNs effectively alleviates acute colitis by promoting the expansion of CD4+CD8+ T cells [2]. Another study indicates that microneedles loaded with *Portulaca oleracea* L.-derived vesicles significantly reduce the protein expression of nuclear factor-κB (NF-κB) and stimulator of interferon genes (STING) in diseased tissues, highlighting their potential for skin anti-inflammation [8].

While research on animal ELNs has advanced considerably, studies on plant-derived ELNs, including extraction standardization, component analysis, and functional mechanisms, are still in their early stages. In particular, the differences in physicochemical properties, active ingredient, and biological functions of ELNs from *Portulaca oleracea* L. in different processing states, such as fresh and dried materials, have not yet been thoroughly investigated [1].

In traditional Chinese herbal medicines, drying is an essential step in the manufacturing of herbal preparations and functional foods, as well as in long-term storage. Most Chinese herbal medicines are sold as dried products. Studies have shown that fresh plant materials retain more medicinal and nutritional components while preventing the loss of active compounds during processing [9]. For example, fresh *Portulaca oleracea* L. extract showed stronger hypoglycemic effects than dried materials in insulin-resistant HepG2 cell models and streptozotocin-induced C57BL/6J diabetic mouse models [7]. This difference is attributed to the degradation of heat-sensitive components, such as certain miRNAs and polyphenols, while drying, as well as structural changes in alkaloids [6]. However, due to preservation challenges, fresh herbal materials are less frequently utilized in practice.

Current research on plant-derived ELNs primarily uses fresh materials for extraction [10]; however, some studies use dried materials soaked in solution [11]. The effect of fresh versus dried materials from the same plant on the bioactive components of ELNs has not been extensively studied. However, it directly affects their biological activity and clinical potential [3]. To address this, ELNs were separately extracted from fresh (F-PELNs) and dried (D-PELNs) *Portulaca oleracea* L. and compared for composition in this study. Multi-omics analysis was performed to compare the metabolites, lipids, and proteins in PELNs from both sources. This study lays the groundwork for further research on *Portulaca oleracea* L. and PELNs by directly revealing the compositional differences between ELNs derived from fresh and dried materials. It also provides a reference for selecting fresh or dried materials for future plant-derived ELN extraction.

## 2. Results

### 2.1. Morphological Identification of Portulaca oleracea L.

**Figure 1:**
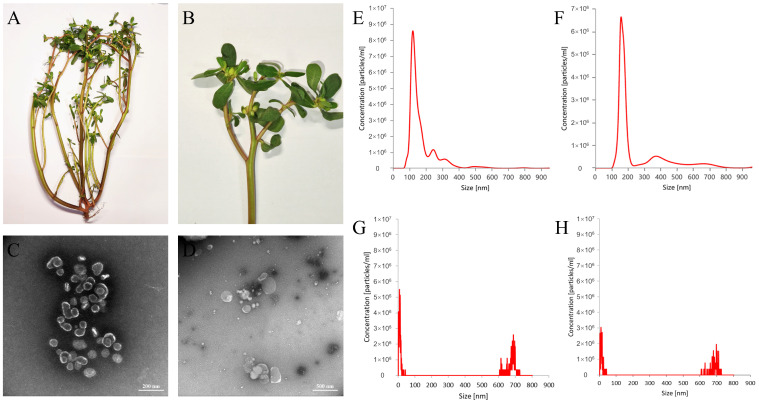
Morphological identification of *Portulaca oleracea* L. and characterization of PELNs: (**A**) habit of the whole plant, (**B**) close-up view, (**C**,**D**) TEM structural diagram of D-PELNs and F-PELNs, (**E**,**F**) particle size distribution diagram of D-PELNs and F-PELNs determined by NTA, and (**G**,**H**) particle size distribution diagram of D-PELNs and F-PELNs determined by NTA after treatment with 1% Triton X-100.

Morphological examination of the whole plant, *Portulaca oleracea* L., showed that the stems were creeping or ascending, fleshy, cylindrical, smooth, glabrous, and often yellowish-brown, with distinct internodes. The stems measured 30 cm in length and 2 mm in diameter and readily broke to reveal a white, pithy core. The leaves were greenish-brown, thick and fleshy, arranged alternately or nearly oppositely, and were obovate, spatulate, or cuneate in shape. The apex was obtuse or slightly concave, while the base tapered gradually into a short petiole or was nearly sessile. Each leaf was 1–2.5 cm in length and 0.5–1.5 cm in width and had entire margins. The veins were inconspicuous, and the flowers were small and sessile, usually clustered in groups of 3–5 at branch tips or leaf axils. The flowers had five yellow obovate petals. The calyx consisted of two green sepals. The capsule was conical, about 5 mm long, and dehiscent at maturity, and contained numerous minute, reniform, black or dark brown seeds with tuberculate surfaces (Figure 1A,B).

### 2.2. Physical Characterization of PELNs

#### 2.2.1. Appearance and Morphology

Transmission electron microscopy (TEM) validated the morphological characteristics of the isolated D-PELNs and F-PELNs (Figure 1C,D). Microscopic images displayed abundant cup-shaped or spherical vesicles in both samples, with distinct lipid bilayer membranes at the vesicle edges, consistent with the typical ultrastructural features of ELNs. The vesicle sizes ranged from 80 to 400 nm, and F-PELNs were usually larger than D-PELNs, consistent with the nanoparticle tracking analysis (NTA) results below. Protein aggregates or cellular debris were rarely seen, indicating high sample purity. These findings are consistent with internationally recognized ELN size standards [12], corroborate the efficacy of the separation protocol, and provide crucial morphological evidence of successful ELN isolation and identification.

#### 2.2.2. Particle Size and Zeta Potential

The particle sizes of D-PELNs and F-PELNs ranged from 80 to 400 nm, with a dominant peak near 180 nm (Figure 1E,F), consistent with the reported characteristics of plant-derived ELNs [13]. NTA showed average particle sizes of 139 nm and 186 nm, with particle concentrations of 5.89 × 10^8^ particles/mL and 4.51 × 10^8^ particles/mL, respectively. Treatment with 1% Triton X-100 resulted in a significant reduction in the dominant nanoparticle peak in both D-PELNs and F-PELNs (Figure 1G,H), consistent with the disruption of lipid bilayer structures. As demonstrated in studies of plant-derived nanovesicle, Triton X-100 treatment leads to the disappearance or splitting of the main particle size peak, indicating vesicle destruction [14]. These characteristics, combined with the average zeta potential measurements (D-PELNs: −16.015 ± 0.335 mV; F-PELNs: −6.29 ± 0.19 mV), confirmed that both types of ELNs were typical vesicular ELNs that met the standard features of ELNs [15,16].

### 2.3. Metabolite Determination

#### 2.3.1. Analysis of PELN Metabolite Composition

**Figure 2:**
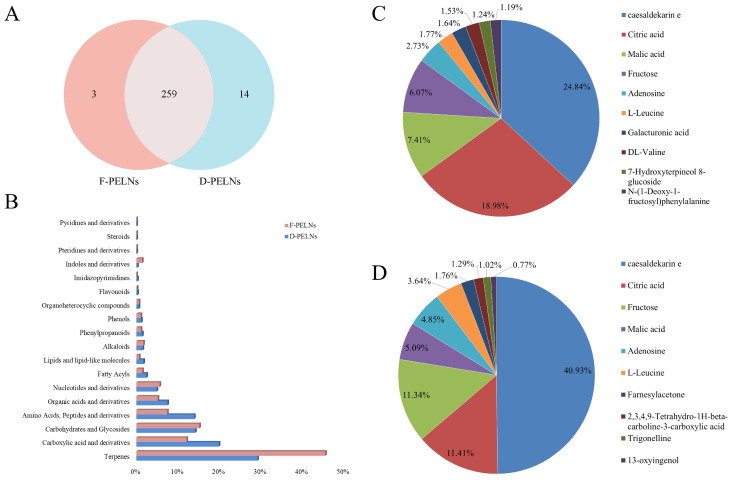
Classification and Top 10 ranking of bioactive compounds by content: (**A**) Venn diagram of bioactive compounds in D-PELNs and F-PELNs, (**B**) content distribution profile of bioactive compounds by category, (**C**) Top 10 bioactive compounds by content in D-PELNs, and (**D**) Top 10 bioactive compounds by content in F-PELNs.

Non-targeted metabolomics analysis identified 273 metabolites in D-PELNs and 262 metabolites in F-PELNs. A Venn diagram showed that 94.87% of the metabolites were common between both groups (Figure 2A). The core metabolites in both D-PELNs and F-PELNs were terpenoids (e.g., cesaldekarin e) and carboxylic acid derivatives (e.g., citric acid and malic acid). The metabolite classification distributions for D-PELNs and F-PELNs are depicted in Figure 2B. The top 10 metabolite distribution profiles (Figure 2C,D) showed that caesaldekarin e and citric acid were the two most abundant metabolites in both D-PELNs and F-PELNs. The top five components in D-PELNs were caesaldekarin e (24.84%), citric acid (18.98%), malic acid (7.41%), fructose (6.07%), and adenosine (2.73%), whereas, the top five metabolites in F-PELNs were caesaldekarin e (40.93%), citric acid (11.41%), fructose (11.34%), malic acid (5.09%), and adenosine (4.85%).

#### 2.3.2. Analysis of Relative Content Differences in PELN Metabolites

**Figure 3:**
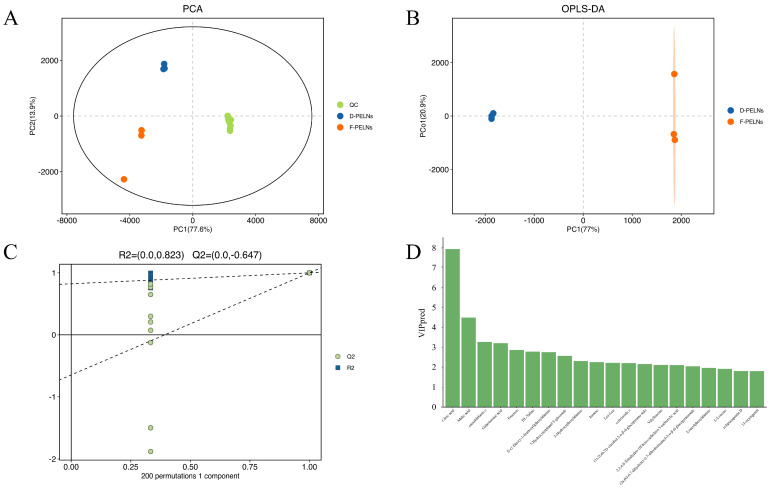
Multivariate Statistical Analysis of D-PELNs and F-PELNs: (**A**) PCA plots of bioactive compounds, (**B**) OPLS-DA plots of bioactive compounds, (**C**) 200-permutation validation plot for OPLS-DA model, (**D**) screening of Top 20 bioactive compounds based on VIP > 1.

To assess the differences in metabolites between D-PELNs and F-PELNs, a multivariate statistical analysis was conducted. The results of the principal component analysis (PCA) are shown in Figure 3A, where the tight clustering of quality control (QC) samples indicates good instrument stability [17]. The D-PELN and F-PELN groups showed partial separation; therefore, supervised orthogonal partial least squares discriminant analysis (OPLS-DA) was applied to improve group discrimination. Figure 3B shows that D-PELN and F-PELN samples were completely separated, confirming significant differences in the metabolic profiles of ELNs derived from dried and fresh materials. This model was validated using seven-fold cross-validation and 200 response permutation tests (RPTs), as shown in Figure 3C. The permutation test findings (R^2^ = 0.823, Q^2^ = −0.647) satisfy the Q^2^ < 0 threshold requirement [18], effectively ruling out the risk of overfitting the model. The top 20 compounds with variable importance in projection (VIP) scores greater than 1 are shown in Figure 3D.

Differential metabolites were identified using a multi-criteria evaluation approach to understand the differences in the active ingredient content between D-PELNs and F-PELNs. This approach used standardized metabolite data and considered the VIP score from multivariate analysis, the *p*-value from Student’s *t*-test, and the fold change (FC). Differential metabolites were screened using the following criteria: *p*-value < 0.05, FC ≥ 1.2, or FC ≤ 1/1.2. Volcano plots were generated to enable the clear observation and selection of differential metabolites between the two groups. Based on the volcano plot results, the top 50 compounds with the greatest relative differences were selected for heatmap analysis, while the top 20 compounds were used for correlation analysis.

**Figure 4:**
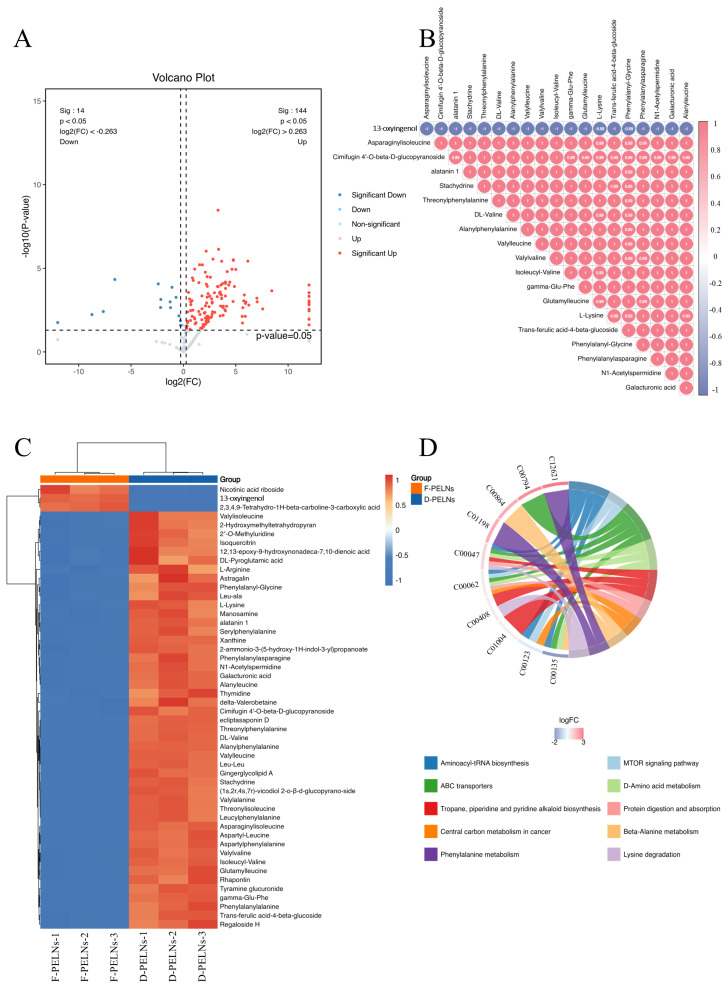
Screening differential metabolites between D-PELNs and F-PELNs: (**A**) volcano plots of differential metabolites, (**B**) correlation analysis of differential metabolites, (**C**) heatmap of differential metabolites, (**D**) KEGG enrichment analysis and chord diagram of differential metabolites.

A volcano plot of differentially expressed metabolites is shown in Figure 4A. Compared to the F-PELN group, 144 metabolites were significantly enriched, whereas 14 metabolites were depleted in the D-PELN group (*p*-value < 0.05, FC ≥ 1.2, or FC ≤ 1/1.2). A complete list is presented in the Supplementary Table S1. Correlation analysis of the top 20 metabolites with the highest VIP scores revealed a positive correlation among various dipeptides, amino acid derivatives, and specialized metabolites. In contrast, 13-oxyingenol showed a negative correlations with all these co-expressed metabolites. The heatmap (Figure 4C) displayed distinct hierarchical clustering, with D-PELNs (corresponding to the red sample groups) enriched in multiple dipeptides (e.g., phenylalanyl-glycine, Leu-Ala), as well as substances like cimifugin 4′-O-beta-D-glucopyranoside, L-lysine, and trans-ferulic acid-4-beta-glucoside, whereas F-PELNs, corresponding to the blue sample groups, are enriched in metabolites including nicotinic acid riboside, 13-oxyingenol, and 2,3,4,9-tetrahydro-1H-beta-carboline-3-carboxylic acid.

To further elucidate the mechanisms underlying metabolic pathway alterations, KEGG pathway enrichment analysis was conducted on all differentially expressed metabolites (*p* ≤ 0.05). The top 10 pathways with listHits > 1 and the lowest *p*-values were chosen for the chord diagram visualization. Figure 4D illustrates that multiple metabolic pathways are involved in differential regulation, among which ABC transporters, phenylalanine metabolism, as well as tropane, piperidine, and pyridine alkaloid biosynthesis are significantly enriched. Based on the logFC color gradient (−2 to 3), metabolites displayed in red are highly expressed, particularly in pathways such as protein digestion and absorption, tropane, piperidine, and pyridine alkaloid biosynthesis. In contrast, metabolites shown in blue exhibit relatively low expression levels, especially in pathways like aminoacyl-tRNA biosynthesis.

### 2.4. Lipid Determination

**Figure 5:**
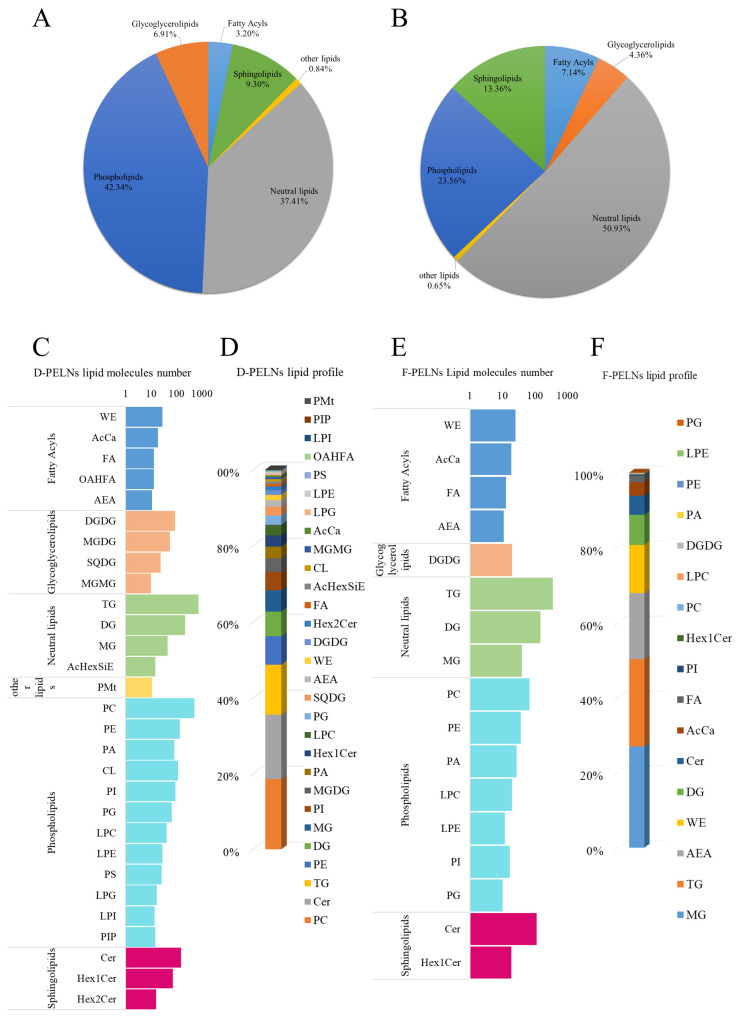
Analysis of lipid composition of D-PELNs and F-PELNs using lipidomics: (**A**) lipid composition classification in D-PELNs, (**B**) lipid composition classification in F-PELNs, (**C**) number distribution of lipid components in D-PELNs, (**D**) content distribution of lipid components in D-PELNs, (**E**) number distribution of lipid components in F-PELNs, (**F**) content distribution of lipid components in F-PELNs.

The lipid compositions of D-PELNs and F-PELNs are shown in Figure 5A and Figure 5B, respectively. In the D-PELNs, 2721 lipids were identified, with phospholipids (42.34%), neutral lipids (37.41%), and sphingolipids (9.30%) being the most abundant classes. In F-PELNs, 1078 lipids were identified, with neutral lipids accounting for the majority (50.93%), followed by phospholipids (23.56%) and sphingolipids (13.36%).

Figure 5C–F shows the number and content profiles of lipid components. In D-PELNs, the top five components by number were triglycerides (TG), phosphatidylcholine (PC), diglycerides (DG), ceramides (Cer), and phosphatidylethanolamine (PE). The top five components by content were phosphatidylcholine (PC), ceramide (Cer), triglycerides (TG), phosphatidylethanolamine (PE), and diglycerides (DG), with contents of 18.48%, 17.02%, 13.15%, 7.49%, and 6.52%, respectively. In F-PELNs, the top five components by number were triglycerides (TG), diglycerides (DG), ceramides (Cer), phosphatidylcholine (PC), and monoglyceride (MG). The top five components by content were monoglyceride (MG), triglyceride (TG), N-acetylethanolamine (AEA), wax esters (WE), and diglycerides (DG), with contents of 27.03%, 23.33%, 17.60%, 12.77%, and 8.05%, respectively.

### 2.5. Protein Determination

**Figure 6:**
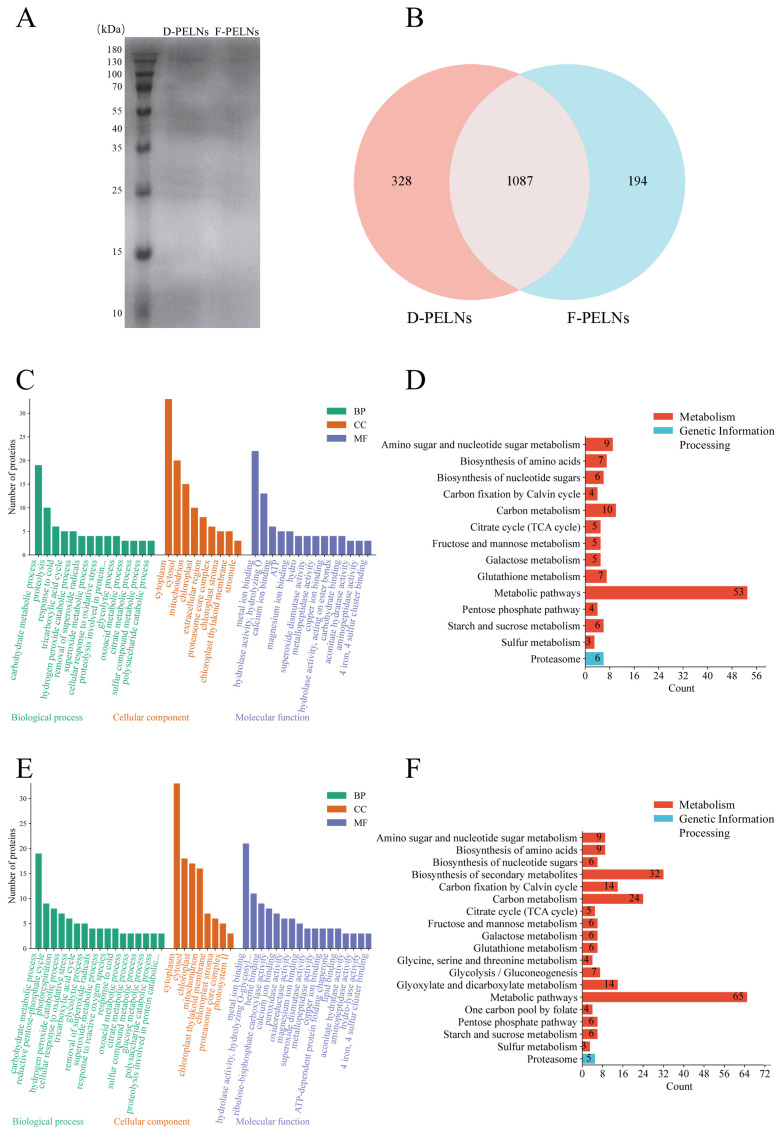
Analysis of the protein composition and function of D-PELNs and F-PELNs: (**A**) total protein analysis of D-PELNs and F-PELNs using SDS-PAGE, (**B**) Venn diagram of total proteins in D-PELNs and F-PELNs using proteomics, (**C**) GO function analysis of D-PELN proteins, (**D**) KEGG function analysis of D-PELN proteins, (**E**) GO function analysis of F-PELN proteins, (**F**) KEGG function analysis of F-PELN proteins.

The BCA assay (*n* = 3) showed average protein concentrations of 1.28 mg/mL in D-PELNs and 0.69 mg/mL in F-PELNs. SDS-PAGE analysis revealed a broad protein distribution, with bands mostly concentrated between 35 and 55 kDa (Figure 6A).

Proteomics analysis using UniProt identification detected 1415 proteins in D-PELNs and 1281 in F-PELNs, with 1087 shared proteins (76.82%) (Figure 6B). GO functional analysis (Figure 6C,E) demonstrated high enrichment in pathways linked to the cytoplasm, cytosol, metal ion binding, carbohydrate metabolism, chloroplasts, and mitochondria. These pathways were associated with energy metabolism, signal transduction, and cellular structure, collectively supporting cellular homeostasis. KEGG clustering indicated significant enrichment in metabolism and genetic information processing, with metabolism taking precedence (Figure 6D,F). D-PELNs showed higher enrichment in carbon metabolism (Figure 6D), whereas F-PELNs were more enriched in the biosynthesis of secondary metabolites (Figure 6F). Both groups showed minimal enrichment in Genetic Information Processing, with only the proteasome pathway showing moderate enrichment.

## 3. Discussion

Plant-derived ELNs have received attention in the biomedical field due to their high biosafety, low immunogenicity, strong absorption efficiency, and targeted delivery capability. In this study, dried and fresh *Portulaca oleracea* L. exosome-like nanoparticles (D-PELNs and F-PELNs) were separated and purified using differential centrifugation, ultracentrifugation, and membrane filtration. This method, which is regarded as the gold standard for plant-derived ELN isolation [19,20,21], yielded nanoparticles with characteristic cup-shaped structures encapsulated by lipid bilayer membranes, as validated by TEM. This study systematically analyzed the composition of dried and fresh ELNs from *Portulaca oleracea* L., bridging the gap in comparing ELN components from the same plant in different states and addressing the limitations of previous studies that focused on fresh materials. Fresh raw materials offer advantages in preserving active substances, but spoil quickly and require immediate processing. On the contrary, dried materials were easier to store and process in bulk, and they also produced ELNs that were structurally intact and functionally preserved. This significantly reduces the application limitations of fresh materials while also providing a reliable strategy for the efficient use of dried medicinal plant resources. In selecting the drying method, we referred to the common practice in traditional Chinese medicine processing where hot-air drying is widely employed as a standard approach for herb preservation [22]. Based on this convention, our study utilized hot-air drying at 45 °C. However, this method likely contributed to the observed reduction in volatile terpenoids in D-PELNs compared to F-PELNs. This aligns with findings in Zingiber officinale, where vacuum freeze-drying was superior to hot air drying in retaining thermosensitive compounds, such as gingerols and flavonoids [23]. Future studies comparing freeze-drying, vacuum drying, and heat drying for *Portulaca oleracea* L. could optimize the preservation of its unique bioactive profile, as research shows that vacuum and microwave drying are more effective at preserving key bioactive molecules in purslane compared to conventional methods [24,25]. These findings also set the foundation for the large-scale preparation and clinical translation of PELNs. In addition, a limitation of our isolation method is the potential co-isolation of non-vesicular contaminants. Future work will employ advanced purification strategies, such as coupling ultracentrifugation with density gradient centrifugation or size-exclusion chromatography, to obtain PELN preparations of higher purity for more definitive functional attribution [26].

Comprehensive metabolite detection was utilized to scan and analyze metabolic profiles, making it ideal for studying complex, low-abundance nanovesicles, such as plant-derived ELNs. UHPLC-Q-TOF-MS analysis demonstrated that the metabolic components of dried and fresh PELNs overlapped extensively while also differing. The relative content of caesaldekarin e was lower in the D-PELNs (24.84%) than in the F-PELNs (40.93%). This difference may be due to the loss of volatile terpenoids during high-temperature drying, whereas F-PELNs extracted from fresh material retain these constituents [27]. The relative increase in non-volatile acids, such as citric acid, in D-PELNs may have been caused by water loss and the release of endogenous substances from disrupted cell structures [9]. Differential metabolite analysis showed that F-PELNs were enriched in metabolites such as 2,3,4,9-tetrahydro-1H-beta-carboline-3-carboxylic acid. In contrast, D-PELNs accumulated dipeptides and amino acid derivatives, including alanylphenylalanine, threonylphenylalanine, and phenylalanyl-glycine, which could indicate drying-induced proteolysis and membrane damage [9], implying that processing methods influence metabolic states [28]. KEGG pathway analysis showed that metabolites with higher abundance in D-PELNs were primarily enriched in pathways related to protein digestion and absorption, as well as tropane, piperidine, and pyridine alkaloid biosynthesis. This pattern corresponds with the observed accumulation of various dipeptides in D-PELNs, implying a potential relationship between the drying process and protein degradation processes, as well as the activation or preservation of specific alkaloid biosynthesis pathways [29,30]. However, given the widespread use of drying in practical applications, protective agents or optimal drying conditions should be investigated to limit the loss of heat-sensitive components [9]. Future research should integrate targeted metabolomics with in vivo models to confirm the significance of specific metabolites and optimize delivery strategies.

Lipids constitute both the structural backbone and active carriers of plant-derived ELNs, and their compositional dynamics have a direct impact on vesicle stability and biological functions. In this study, non-targeted lipidomics revealed that drying treatment profoundly altered the lipid composition of PELNs from *Portulaca oleracea* L. D-PELNs exhibited greater lipid diversity than F-PELNs, whereas F-PELNs were enriched with functional neutral lipids. This disparity likely stems from drying-induced membrane lipid rearrangement, oxidative stress responses, and radical reactions [31,32]. Specifically, the significant enrichment of PC (18.48%) and Cer (17.02%) in D-PELNs may suggest an adaptive response to maintain vesicle membrane integrity during drying stress, as well as lipid reprogramming observed in plants under drought or heat stress [33,34]. In contrast, F-PELNs had higher levels of neutral and signaling lipids, such as MG, TG, and AEA, indicating that fresh extraction better preserved native bioactive lipids. Notably, AEA functions as an endogenous cannabinoid receptor agonist with neuromodulatory properties in plant-derived ELNs [35], whereas MG regulates inflammatory pathways via PPARγ [36,37,38]. The accumulation of WE in F-PELNs may also contribute to enhanced membrane integrity and reduced lipid degradation [39]. Although D-PELNs exhibited broader lipid diversity, future studies will incorporate *Portulaca oleracea* L. samples from different geographical regions to verify the universality of these findings.

Proteins are important functional carriers in plant-derived ELNs, and they participate in processes such as signal transduction, stress response, and host–microbe interactions [40,41]. In this study, SDS-PAGE revealed that the protein bands in both samples were mainly distributed between 35 and 55 kDa. Proteomic analysis demonstrated that 76.82% of proteins were shared between the two groups. These findings suggest that, while processing methods may cause some protein differences, the core composition of ELNs is highly conserved. This finding is consistent with previous reports showing structural stability and functional preservation in plant-derived ELNs [3]. GO and KEGG analyses revealed that exosomal proteins from both groups were widely localized to intracellular structures, such as the cytoplasm and mitochondria. Functionally, they were enriched in pathways such as carbohydrate metabolism and metal ion binding, suggesting possible involvement in material transport and cellular homeostasis. D-PELNs exhibited stronger enrichment in carbon metabolism pathways, whereas F-PELNs were more enriched in secondary metabolite synthesis pathways. This suggests that fresh material may better sustain secondary metabolic enzyme activity, although drying may increase the proteins involved in basal metabolism [42]. Gene information processing showed relatively low enrichment, with only the proteasome pathway being slightly represented. This suggests that PELNs are largely responsible for material metabolism and transport, rather than genetic information processing. This finding aligns with the conclusions of most plant-derived ELN studies, which focus on antioxidant stress and moderate regulation [43,44].

Compared to ELNs from other plant species, PELNs display distinctive features. Unlike ginger-derived ELNs, which contain high levels of specific lipids such as phosphatidic acid [43,45], PELNs are characterized by a high abundance of terpenoids (e.g., caesaldekarin e) and specific carboxylic acids. In addition, the presence of unique lipid species, such as high levels of AEA—a lipid mediator with multiple biological functions in F-PELNs—is rarely reported in ELNs from sources like grapefruit or lemon [43]. Collectively, these comparative results underscore the unique lipid and metabolite composition of PELNs, highlighting their potential as promising candidates for specialized bioactivities and cross-kingdom communication.

Based on the data presented above, D-PELNs may be more suitable for studying plant basal metabolic reprogramming and stress-related products, whereas F-PELNs show greater potential for carrying plant secondary metabolic molecules for biofunctional research or therapeutic applications. However, this study lacked functional validation of differential metabolites as well as data supporting enzyme activity in non-metabolic pathways. Non-targeted methods fail to distinguish certain key isomeric lipids, and protein analysis does not establish the specific functions of differentially expressed proteins. Future studies should integrate cellular or animal models with mechanistic investigations by combining targeted lipidomics and spatial imaging mass spectrometry (e.g., MALDI-IMS) to determine the localization of key lipids within vesicular membranes and their involvement in signaling and transport. Therefore, investigating how different processing methods affect PELN bioactivity will aid in optimizing preparation protocols and enhancing medicinal development, advancing their usage in plant compound delivery and natural drug research [46,47].

## 4. Materials and Methods

### 4.1. Materials and Reagents

Fresh *Portulaca oleracea* L. (commercially available, Shandong, China); BCA protein concentration assay kit, phosphate-buffered saline, horseradish peroxidase-labeled goat anti-rabbit IgG (H + L) (Beyotime Biotechnology Co., Ltd., Shanghai, China); phosphotungstic acid (Merck, Darmstadt, Germany); PageRuler Prestained Protein Ladder (ThermoFisher Scientific, Waltham, MA, USA); methanol, formic acid, ethanol, acetonitrile, and isopropanol (chromatography-grade, Shanghai Aladdin Biochemical Technology Co., Ltd., Shanghai, China).

### 4.2. Identification of Portulaca oleracea L.

In this study, *Portulaca oleracea* L. from Shandong Province was selected as the material. Fresh roots, stems, leaves, and flowers were morphologically examined and identified by Associate Professor Wang Mengyue of Shanghai Jiao Tong University as the aerial parts of *Portulaca oleracea* L., a member of the Portulacaceae family.

### 4.3. Extraction and Separation of PELNs

Fresh *Portulaca oleracea* L.-derived Exosome-Like Nanoparticles (F-PELNs): Approximately 1000 g of fresh *Portulaca oleracea* L. were weighed, washed, and squeezed. Differential centrifugation was performed to remove the fibrous impurities. The juice was centrifuged at 4 °C for 1000× *g* (10 min), 3000× *g* (30 min), 5000× *g* (30 min), and 10,000× *g* (1 h). The supernatant was collected and filtered using a 0.8 μm microporous filter membrane, and centrifuged at 100,000× *g* for 1.5 h at 4 °C. The supernatant was discarded, and the pellet was resuspended in an appropriate amount of pre-chilled PBS before being centrifuged again at 100,000× *g* for 2 h to obtain the gel pellet. The gel was resuspended in pre-chilled PBS by sonication. The resuspension was filtered sequentially through 0.8 μm and 0.45 μm microporous filter membranes, yielding 30 mL of F-PELNs, which were stored at 4 °C for future use.

Dried *Portulaca oleracea* L.-derived Exosome-like Nanoparticles (D-PELNs): Fresh *Portulaca oleracea* L. was processed according to the Chinese Pharmacopoeia, 2020 edition. Approximately 1000 g of fresh *Portulaca oleracea* L. was weighed, washed, slightly moistened, and cut into segments. The plant material was then dried using a hot air oven at 45 °C for 48 h. The dried material was ground into a powder. The powder was mixed with PBS in a ratio of 1:10 (*w*/*v*) and refrigerated overnight at 4 °C. The filtrate was subjected to differential centrifugation, ultracentrifugation, membrane filtration, and resuspension of PBS as described for fresh *Portulaca oleracea* L. A total of 30 mL of D-PELNs was obtained and stored at 4 °C for future use.

Both sample types were analyzed in three biological replicates (*n* = 3). Each replicate was derived from a pooled sample of multiple individual plants from the same batch.

### 4.4. Physical Characterization of PELNs

#### 4.4.1. Appearance and Morphology

The hydrophilic-treated copper mesh was treated with a glow discharge instrument (Shenzhen Super Instruments Co., Ltd., Shenzhen, China) to enhance the adsorption of PELN samples onto the carbon film. The copper mesh was held with self-locking tweezers and suspended over a Petri dish. Six microliters of the sample were added to a copper mesh and allowed to stand for 5 min. The excess liquid was removed using filter paper, and one drop of ultrapure water was added to the copper mesh. After 10 s, the water was drained. This process was repeated three times to eliminate the salt components. Subsequently, ten microliters of phosphotungstic acid were added to the copper mesh. After 1 min of staining, the excess solution was blotted with filter paper. The copper mesh was dried under a heating lamp and then placed in a 120 kV TEM (Thermo Fisher Scientific, Waltham, MA, USA) for analysis. The morphological structures of D-PELNs and F-PELNs were examined, photographed, and recorded.

#### 4.4.2. Particle Size and Zeta Potential

Forty microliters of D-PELNs was divided into two equal portions. One portion (20 μL) was lysed using 1% Triton. The mixture was vortexed for 2 min and refrigerated at 4 °C for 20 min to obtain the D-PELN lysate. The remaining 20 μL was used as the D-PELN sample. The same procedure was repeated for the F-PELN sample. Four samples were obtained—D-PELNs, D-PELN lysate, F-PELNs, and F-PELN lysate—for subsequent analysis.

NTA: The ZetaView TWIN nanoparticle analyzer (Particle Metrix, Germany) was launched and allowed to warm up. The chamber was cleaned with ultrapure water using a syringe. After self-checking, a 100 nm microsphere standard was injected into the chamber for calibration. The chamber was cleaned again with ultrapure water before loading the sample. Auto-focus was selected, and detection was initiated to assess the nanoparticle concentration, size distribution, and zeta potential of D-PELNs, D-PELN lysate, F-PELNs, and F-PELN lysate.

### 4.5. LC-MS-Based Metabolomics Analysis

Sample Preparation: 1.5 mL of D-PELNs and 1.5 mL of F-PELNs were transferred into centrifuge tubes. ELNs were disrupted by repeated freeze–thaw cycles using liquid nitrogen and a 95 °C metal bath. The total protein concentration in each sample was quantified using a BCA protein concentration assay kit and adjusted to 0.5 mg/mL. To each 400 μL aliquot, 400 μL of 0.01% 2,6-di-tert-butyl-4-methylphenol (BHT) in isopropanol was added, and the mixture was incubated at 75 °C for 15 min. Then, 1.5 mL of chloroform/methanol (1:2, *v*/*v*) and 0.5 mL of chloroform were added, and the mixture was thoroughly vortexed. Next, 0.5 mL of water was added, and the mixture was vortexed again. The mixture was centrifuged at 1000 rpm for 5 min. From the separated phases, 500 μL of the upper aqueous layer and 500 μL of the lower organic layer were transferred to separate tubes and evaporated using a vacuum concentrator. The dried aqueous residue was resuspended in 150 μL of water/methanol (18:7, *v*/*v*), vortexed for 30 s, and sonicated in an ice–water bath for 5 min. After centrifugation at 10,000 rpm for 5 min, 100 μL of the supernatant was transferred to a liquid chromatography vial with an inner liner for further analysis. Three parallel samples were prepared for both D-PELNs and F-PELNs. The QC sample was prepared by pooling equal volumes of the extracts from all samples.

Detection was performed using an ACQUITY UPLC I-Class HF system (Waters, Milford, MA, USA) coupled to a Q Exactive (QE) Plus mass spectrometer (Thermo Fisher Scientific, Waltham, MA, USA).

Chromatographic conditions: An ACQUITY UPLC HSS T3 column (100 mm × 2.1 mm, 1.8 μm; Waters Corporation, Milford, MA, USA) was maintained at 45 °C. Mobile phase A consisted of water with 0.1% formic acid (FA), while mobile phase B consisted of acetonitrile (ACN). The flow rate was set to 0.35 mL/min. Gradient elution program: 0–2 min, 5% B; 2–4 min, 5–30% B; 4–8 min, 30–50% B; 8–10 min, 50–80% B; 10–14 min, 80–100% B; 14–15 min, 100% B; and 15–15.1 min, 100–5% B; 15.1–16 min, 5% B. The injection volume was 2 μL.

Mass spectrometry conditions: Sample mass signals were acquired in both the positive and negative ion scanning modes. The data were collected using data-dependent acquisition (DDA) with a Full MS/dd-MS2 (TOP 8) scanning scheme. The normalized collision energy (NCE) for ion fragmentation was set at 10, 20, and 40. The full scan range was 100–1500 *m*/*z*, with a resolution of 60,000. The dd-MS/MS resolution was set to 15,000. The ionization voltages were 3.8 kV in the positive mode and 3.2 kV in the negative mode. The capillary temperature was maintained at 320 °C.

After raw data acquisition, compounds were identified using Progenesis QI 2.3 software (Waters, Milford, MA, USA) and a proprietary local database (Shanghai OE Biotech Co., Ltd., Shanghai, China), based on retention time, MS1 precise mass, MS2 fragments, and isotope distributions. Screening was conducted on the active components identified in the samples using Progenesis QI software and a proprietary local database. The screening criteria were as follows: retention time deviation < 0.2 min; MS1 mass error < 5 ppm; MS/MS spectrum similarity > 80%; and isotope distribution matching with mSigma < 20.

### 4.6. LC-MS-Based Lipidomic Analysis

Sample Preparation: Briefly, 500 μL of the lower organic layer obtained from D-PELNs and F-PELNs, as described in Section 2.5, was transferred to a new centrifuge tube and evaporated in a vacuum concentrator. The residue was resuspended in 300 μL of water/methanol (1:1, *v*/*v*), vortexed for 30 s, and sonicated in an ice–water bath for 5 min. After centrifugation at 10,000 rpm for 5 min, 100 μL of the supernatant was transferred to a liquid chromatography vial with a liner tube. Three parallel samples were prepared for each sample type. The QC sample was prepared by pooling equal volumes of extracts from all samples.

Detection was performed using a Vanquish UHPLC system (Thermo Fisher Scientific, Waltham, MA, USA) equipped with a dual-channel pump, vacuum degasser, auto-sampler, and column oven, coupled to a QE Plus mass spectrometer (Thermo Fisher Scientific, Waltham, MA, USA).

Chromatographic conditions: An ACQUITY UPLC BEH C18 column (100 × 2.1 mm, 1.7 μm) was maintained at 60 °C. Mobile phase A consisted of acetonitrile/water (60:40, *v*/*v*) with 10 mM ammonium formate and 0.1% formic acid, and mobile phase B consisted of isopropanol/acetonitrile (90:10, *v*/*v*) with 10 mM ammonium formate and 0.1% formic acid. The flow rate was set to 0.4 mL/min. Gradient elution program: 0–2 min, 40–43% B; 2–2.1 min, 43–50% B; 2.1–12 min, 50–54% B; 12–12.1 min, 54–70% B; 12.1–18 min, 70–99% B; 18–18.1 min, 99–40% B. The injection volume was 1 μL.

Mass spectrometry conditions: Mass spectrometry signals were collected using both positive and negative ion scanning modes. The data were acquired using a Full MS/dd-MS2 (TOPN) scanning method. The NCE was set at 15, 30, and 45 for ion fragmentation. The full scan range was 150–2000 *m*/*z*, with a resolution of 70,000. Automatic gain control (AGC) was set at 10^6^, with a maximum ion injection time (IT) of 100 ms. The dd MS/MS resolution was 17,500. The spray voltages were 3.2 kV in the positive mode and 3.0 kV in the negative mode. The capillary temperature was set to 320 °C.

After raw data acquisition, Lipidsearch 4.2 software (ThermoFisher Scientific, Waltham, MA, USA) was used to search for and identify the detected lipids. Subsequently, Xcalibur 3.0 software (ThermoFisher Scientific, Waltham, MA, USA) was used to identify and extract the detected lipid ion pair information.

### 4.7. Protein Determination

Sample Preparation: A volume of 1 mL of D-PELNs and F-PELNs was transferred into centrifuge tubes. The ELNs were disrupted by repeated freeze–thaw cycles using liquid nitrogen and a 95 °C metal bath. The total protein concentration in each sample was quantified using a BCA protein concentration assay kit.

#### 4.7.1. Protein Quantification and SDS-PAGE Analysis

All samples were uniformly diluted to a total protein concentration of 1 mg/mL, as per the BCA assay results. After the addition of 5× loading buffer, the samples were boiled to denature the proteins. Proteins from D-PELNs and F-PELNs were separated by standard SDS-PAGE, using 5% stacking gel and 10% separating gel, until the rainbow marker band was fully resolved. Following separation, the stacking gel was removed, and the resolving gel was cut into appropriately sized pieces. The gel plates were stained with Coomassie Brilliant Blue and destained using a destaining solution before being developed, observed, photographed, and documented.

#### 4.7.2. LC-MS-Based Proteomic Analysis

All samples were diluted to a uniform total protein concentration of 200 μg/mL, as per the BCA assay results. A total of 2 μL of 1 M dithiothreitol (DTT) was added to each 200 μL sample, and reduction was performed at 37 °C in a water bath for 1 h. Subsequently, 10 μL of freshly prepared 1 M iodoacetamide was added, and alkylation was performed at room temperature in the dark for 40 min. The solvent was replaced with 50 mM NH_4_HCO_3_ using a 10 kDa ultrafiltration membrane. Next, trypsin solution (0.5 ng/μL) was added, and the samples were digested overnight at 37 °C. Following digestion, the peptides were desalted using a C18 desalting column before mass spectrometry analysis. Three parallel samples were prepared for each sample type. The QC sample was prepared by pooling equal volumes of the extracts from all samples.

Detection was performed using an Easy-nLC 1200 system coupled to a Q-Exactive Plus mass spectrometer (Thermo Fisher Scientific, Bremen, Germany).

Chromatographic conditions: A C18 reversed-phase analytical column (75 mm × 20 cm × 3 μm) was used. Mobile phase A consisted of water with 0.1% formic acid, and mobile phase B consisted of acetonitrile/water (80:20, *v*/*v*) solution with 0.1% formic acid. The flow rate was set to 300 nL/min. Gradient elution program: 0–2 min, 2–6% B; 2–95 min, 6–20% B; 95–107 min, 20–32% B; 107–108 min, 32–100% B; 108–129 min, 100% B. The injection volume was 1 μL.

Mass spectrometry conditions: Sample signals were acquired in the positive ion scanning mode. The NCE was set at 28 for ion fragmentation. The full scan range was 350–1800 *m*/*z*, with a resolution of 70,000. AGC was set to 3e6 with a maximum IT of 50 ms. The dd MS/MS resolution was 17,500. The spray voltage was 2.3 kV, and the capillary temperature was 275 °C. The top 20 most abundant peptides were selected for secondary fragmentation. Fragmentation was achieved by High Collision Dissociation (HCD). Ions with charges of 1, 7, 8, and greater than 8 were excluded from the study. The dynamic exclusion time was set to 30 s.

Data Processing: The raw LC-MS/MS data files were analyzed using Proteome Discoverer version 2.4 (Thermo Fisher Scientific, Bremen, Germany). Fixed modifications were set to N-acetylation (C), while variable modifications included oxidation (M), deamidation (N, Q), and N-terminal acetylation (C). The initial mass tolerances for the precursor and fragment ions were set at 10 ppm and 0.02 Da, respectively. A total of two trypsin cleavage sites were permitted. Peptide identification was filtered to include only proteins detected with at least two unique peptides and a false discovery rate (FDR) of less than 1%. Label-free quantification was performed using unique peptides via the Top3 method to calculate protein abundance. The corresponding parent ions were extracted from the MS scans to obtain label-free quantitative values (peak intensity) for unique peptides. Quantified proteomic data were normalized using the protein abundance-based approach, and ANOVA was performed at the individual protein level.

### 4.8. Statistical Analysis

For each omics dataset, compounds with a coefficient of variation (CV) > 30% in the QC sample group and an average peak area < 1000 were excluded. Samples with an intra-group missing rate > 50% were eliminated, and missing values were replaced with half of the minimum positive value. The data were then median-normalized, and the resulting standardized data were used for subsequent statistical analyses.

Standardized data for the active ingredients were analyzed using multivariate statistical analysis with SIMCA 14.1 software (Umetrics, Sweden). Standardized protein data were analyzed using the online database David (https://davidbioinformatics.nih.gov/). The analyses included GO enrichment (ranked by protein count and categories with ≥2 proteins per pathway) and KEGG pathway analysis (ranked by gene enrichment count). Enriched pathway diagrams were generated using the MicroBioinformatics online platform (https://www.bioinformatics.com.cn/). All other data visualization and statistical analyses were conducted using GraphPad Prism 8.0 (GraphPad, Boston, MA, USA).

## 5. Conclusions

This study successfully isolated and characterized D-PELNs and F-PELNs using differential and ultracentrifugation with membrane filtration. Integrated analyses of metabolomics, lipidomics, and proteomics revealed that drying alters the ELN composition profile, paving the way for the future development and application of *Portulaca oleracea* L.

## Data Availability

All relevant data are included in the article. The raw multi-omics data files generated in this study have been deposited in the Figshare database. The data can be accessed via https://doi.org/10.6084/m9.figshare.30734378 (accessed on 27 November 2025).

## Supplementary Materials

The following supporting information can be downloaded at: https://www.mdpi.com/article/10.3390/molecules30244715/s1. Table S1: Differential bioactive compounds between D-PELNs and F-PELNs.
