# Supplementary material for: Comparative Analysis of the Composition of Exosome-like Nanoparticles from Dried and Fresh Portulaca oleracea L
Source: Molecules. 2025 Dec 9;30(24):4715. doi: 10.3390/molecules30244715 (PMC12736374; doi:10.3390/molecules30244715)
Supplement: Supplementary file 1 [file molecules-30-04715-s001.zip › molecules-3955978-supplementary.pdf]

Table S1 differential bioactive compounds between D-PELNs and F-PELNs

No.	Compounds	Pubchem	FC	P-value
1	Alanylphenylalanine	96814	9.919479058	3.35554E-09
2	Threonylphenylalanine	7010579	10.28811012	7.34589E-07
3	DL-Valine	1182	4.521876368	9.45706E-07
4	Asparaginylisoleucine	56991460	7.112468773	2.85005E-06
5	Galacturonic acid	439215	27.38677507	3.08997E-06
6	Valylvaline	107475	27.39066688	3.36582E-06
7	alatanin 1	/	71.36013455	3.75209E-06
8	Valylleucine	6993117	12.62353651	3.82535E-06
9	N1-Acetylspermidine	496	2.930651995	6.19287E-06
10	gamma-Glu-Phe	111299	3.529115057	6.23149E-06
11	Cimifugin 4'-O-beta-D-glucopyranoside	/	2.121533084	1.11891E-05
12	Isoleucyl-Valine	435949	24.94392022	1.14295E-05
13	Alanyleucine	96801	32.0066579	1.17435E-05
14	Trans-ferulic acid-4-beta-glucoside	/	18.18898088	1.58949E-05
15	Stachydrine	115244	4.276004387	1.71812E-05
16	Glutamylleucine	9856500	4.817627916	3.18828E-05
17	Phenylalanylasparagine	10468817	5.980486466	3.43192E-05
18	L-Lysine	5962	1.820472773	3.7751E-05
19	13-oxyingenol	/	0.010704963	4.66902E-05
20	Phenylalanyl-Glycine	98207	1.920953176	5.3071E-05
21	Valylisoleucine	7010531	16.15170504	6.62377E-05
22	Regaloside H	/	21.58423666	6.70602E-05
23	Manosamine	318324	2.793747672	7.16686E-05
24	2′-O-Methyluridine	102212	16.25975518	7.97588E-05
25	2-Hydroxymethyltetrahydropyran	/	13.53680491	8.17667E-05
26	2,3,4,9-Tetrahydro-1H-beta-carboline-3-carboxylic acid	98285	0.189530805	8.54324E-05
27	Thymidine	5789	4.566406912	8.94421E-05
28	Leu-ala	81721	11.98927919	9.97511E-05
29	Isoquercitrin	/	20.11385769	0.000100685
30	ecliptasaponin D	/	31476485.88	0.000100748
31	Aspartyl-Leucine	332962	4.87499323	0.000120277
32	delta-Valerobetaine	14274897	9.566151967	0.000122195
33	Xanthine	1188	29.43504362	0.000123085
34	Leu-Leu	94244	9.947842471	0.000124437
35	Nicotinic acid riboside	/	0.471765335	0.000137283
36	2-ammonio-3-(5-hydroxy-1H-indol-3-yl)propanoate	144	16.27056318	0.000160518
37	Aspartylphenylalanine	93078	347.7178634	0.00022172
38	Astragalin	5282102	11.45137199	0.000262111
39	L-Arginine	6322	1.398064758	0.000275648

40	Gingerglycolipid A	6450152	10260766.62	0.000288087
41	12,13-epoxy-9-hydroxynonadeca-7,10-dienoic acid	/	10.94812082	0.00029086
42	Valylalanine	6992637	7.828415512	0.000291469
43	Serylphenylalanine	7009597	5.129162392	0.000328899
44	DL-Pyroglutamic acid	/	11.29575047	0.000330895
45	Threonylisoleucine	57281443	12.34545525	0.000389369
46	Rhapontin	637213	56.51117915	0.000395633
47	Phenylalanylalanine	5488196	3.416168864	0.000450173
48	Leucylphenylalanine	6992309	16.0685593	0.000456114
49	(1s,2r,4s,7r)-vicodiol 2-o-β-d-glucopyrano-side	/	5.760517903	0.000459346
50	Tyramine glucuronide	193088	5563668.732	0.000460242
51	japonicumin D	/	551363.8951	0.000484151
52	Ribothymidine	445408	9.390641516	0.000499032
53	Succinyladenosine	20849086	4.836972303	0.000534828
54	2,6-Dimethylquinoline	/	0.614692301	0.000542823
55	atractyloside g	71448957	12.6201552	0.00061126
56	(2R,3R,4S)-2-(6-aminopurin-9-yl)-4-(hydroxymethyl)oxolan-3-ol	/	129.5606366	0.000613971
57	Leucyl-Isoleucine	435718	12.61890868	0.000649177
58	7,8-Didehydro-2-hydroxy-3,7-dimethoxy-17-methylhasubanan-6-one	/	6.476759131	0.000667747
59	Sequoyitol	439990	1.507557502	0.00068064
60	L-Histidine	6274	0.225076438	0.000730607
61	harpagide acetate	/	1.769462208	0.000870778
62	Dehydrophytosphingosine	14757419	54.41025267	0.000886478
63	N-(1-Deoxy-1-fructosyl)leucine	131752244	17547.21438	0.000894717
64	Methyl p-hydroxybenzoate glucoside	/	26.77855339	0.00096862
65	gamma-Glutamylleucine	151023	132.2478883	0.001015152
66	Fucitol	445724	0.416425632	0.001016093
67	Pyroglutamic acid	7405	1.63278117	0.0010167
68	N-(1-Deoxy-1-fructosyl)phenylalanine	101039148	62.61354619	0.001038865
69	Valylphenylalanine	6993119	6.730423793	0.001039527
70	Regaloside E	/	2190979.205	0.001092416
71	Cytidine	6175	4.667672549	0.001178731
72	(3s,6r)-6,7-dihydroxy-6,7-dihydrolinalool-3-o-β-d-glucopyranoside	/	6.713937402	0.001212528
73	Xylobiose	/	60.84913123	0.001259393
74	Corosolic acid	/	1712651.883	0.001381186
75	mucrolidin	/	1.641001737	0.001506496

76	aristoliukine a	/	1365675.422	0.001507013
77	5'-GMP;5'-guanosine monophosphate	135400774	76.18358052	0.001574209
78	3-Hydroxyphenylalanine	/	4.316859909	0.001737751
79	massonivesinol	/	11.75641417	0.001817306
80	Lobetyolin	53486204	14.09160962	0.001850665
81	Inosine	6021	17.55545832	0.001868566
82	Glutamylisoleucine	9813855	5.486615704	0.001989233
83	Acetyllycoposerramine M	/	13.6978154	0.002200956
84	Paniculide B	/	0.221158428	0.002214667
85	griffonin_qt	/	0.42807452	0.002252155
86	1 beta,4 beta,6 beta,11-tetrahydroxyeudesmane	/	51.20169207	0.002274771
87	Phenethylamine glucuronide	191195	3.434300877	0.002617609
88	Morin	5281670	190.4048753	0.002680808
89	6-(alpha-D-Glucosaminyl)-1D-myo-inositol	/	3588637.035	0.002855276
90	γ-glutamyl-alanine	5154674	15.60594212	0.002994766
91	Vomifoliol	5280654	1.875688402	0.003195598
92	4-Carboxycarbostyril	85076	1.622516783	0.003383858
93	Ser-Leu	/	16.14990387	0.003474066
94	PCG	135398570	1603919.956	0.003498218
95	Alanylisoleucine	7408078	2.757542725	0.003800781
96	Thaliporphine	100020	0.004988679	0.003834108
97	3-Coumaric acid	637541	6.902261428	0.00397472
98	Trigonelline	5570	1.324077308	0.004050034
99	dictamnoside,k	/	7.979525992	0.004330169
100	Methylsyringin	15108321	4.203206725	0.004391278
101	2'-O-Methyladenosine	317398	4.115631326	0.00444501
102	Glycyl-Isoleucine	259613	4.001301817	0.004841744
103	Gularic acid	607	6.66452731	0.005772782
104	Songorine	/	3.007844186	0.005804414
105	5'-Methylthioadenosine	439176	0.002348724	0.005869442
106	dendroside e	/	13.86483248	0.006439029
107	L-Leucine	6106	0.744172838	0.006992628
108	Asparaginyl-Phenylalanine	18218184	11.41476958	0.007014977
109	(Z)-isobutyl 2-methylbut-2-enoate	5367806	4.811653211	0.007406137
110	Serylleucine	7015694	60.34370732	0.007414703
111	Phenylalanylthreonine	18218233	5.052512218	0.007456595
112	Esculetin	5281416	1.790253871	0.007462912
113	Glycyl-L-tyrosine	92829	49.1027574	0.007527241
114	deca-6,8-diene-1,3,5-triol	/	6.376684381	0.008262404
115	7-Hydroxyterpineol 8-glucoside	85197858	6.075789182	0.008734931
116	L-Threonic acid	151152	5.220352476	0.008881864
117	N-acetyldopamine	100526	5.05511819	0.009069302

118	Glycyl-Phenylalanine	97415	2.071876624	0.009079237
119	8-NONENOIC ACID	35860	4.290348086	0.009442275
120	Cyclo(Pro-Val)	/	4.906038008	0.009987367
121	6-Methyluracil	12283	1066614.691	0.010607775
122	Aspartyl-Isoleucine	21845165	2.123158487	0.010650455
123	(2s,6 ζ)-3,7-dimethyloct-3(10)-ene-1,2,6,7-tetrol1-o-β-d-glucopyranoside	85242164	5.220624085	0.011080437
124	Fabiatrin	/	356469.1555	0.011231599
125	Apiopaeonoside	/	3.396743556	0.011921281
126	Glycolaldehyde dimer	186078	4.900966691	0.012079458
127	celerioside e	/	4.861404163	0.012936505
128	isololiolide	/	1.602415491	0.013107733
129	Fructose	/	0.819591286	0.013527927
130	Daphnetin	5280569	4.490312477	0.014376287
131	N-Oleoyl glycine	/	1.30667186	0.014502698
132	Rosavin	/	4.619446672	0.015096701
133	Allitol	5780	5.213609075	0.01528674
134	Pipecolinic acid	849	1.336401289	0.01633542
135	2-hex-2-enoxy-6-(hydroxymethyl)oxane-3,4,5-triol	6450053	4.894607156	0.016571014
136	taxezopidin b	/	3.05176E-05	0.017530383
137	Heteroclitin D	/	1.312228293	0.017642144
138	Acanthoside B	443024	2.321173923	0.017789847
139	Prometaphanine	129316922	50.11689741	0.018685908
140	rugosicacid c	101606460	2.177460664	0.020904876
141	Marmesin galactoside	611513	4.40408476	0.021410692
142	D-altrofurano-heptulose-3	/	4.218763586	0.022186269
143	6'-O-beta-D-Glucosylgentiopicroside	/	3.692784658	0.023106435
144	Hydroxycitric acid lactone	/	139284.1746	0.024234805
145	Protocatechuic acid	72	3.734475152	0.024425645
146	Dibenzoylmethane	8433	0.830064989	0.026100929
147	3-cyclohexenylmethanol	15512	1.280503034	0.027871422
148	celephtalide a	/	3.848137032	0.029240841
149	dendronobiloside b	/	3.799527216	0.029852328
150	staphylionoside c	/	3.653728717	0.031679508
151	Sesamol	68289	3.487821791	0.035861145
152	Pantothenic acid	6613	3.512910506	0.036184009
153	Alismoxide	/	1.235198573	0.037253084
154	atractyloside c	71448953	3.133703998	0.037956576
155	3-(2-hydroxyphenyl)propanoate	873	3.431911436	0.038636153
156	Gentiopicroside	88708	1.232800427	0.038680154
157	N2-Methylguanosine	3035422	3.366184012	0.039354084
158	[1,3]Dioxolo[4,5-g]isoquinolin-5(6H)-one	12997610	1.559198631	0.044699491
